# Comparing Pixel Changes and Manual Observations for Mapping Broiler Activity during Dried Black Soldier Fly Larvae (*Hermetia illucens*) Provisioning

**DOI:** 10.3390/ani13132200

**Published:** 2023-07-05

**Authors:** Noa van Leuffen, Allyson F. Ipema, J. Elizabeth Bolhuis

**Affiliations:** 1 Adaptation Physiology Group, Department of Animal Sciences, Wageningen University & Research, 6700 AH Wageningen, The Netherlands; allyson.ipema@wur.nl (A.F.I.); liesbeth.bolhuis@wur.nl (J.E.B.); 2 Department of Research, Aeres University of Applied Sciences, 8251 JZ Dronten, The Netherlands

**Keywords:** broiler welfare, insect feedstuffs, environmental enrichment, video image analysis, activity

## Abstract

**Simple Summary:**

Exercising broilers may reduce health problems as this benefits leg development and limits the time in which broilers’ skin is in contact with dirty litter. Scattering dried Black Soldier Fly Larvae (BSFL) in the whole pen could increase broiler activity. We studied the activity of broilers during dried BSFL feeding periods in more detail to determine how well BSFL provisioning maintains broiler activity. To do so, we assessed broiler activity, at four daily provisioning periods and at different ages. Apart from manual observations, we used a software that automatically measured pixel changes in successive videoframes as a proxy for broiler activity. These pixel changes were positively related to manual observations of activity. Both the automated method and manual observations detected that scattering dried BSFL increased activity. Activity in response to feeding periods was influenced by the time of day and was lowest at 17:00 in manual observations, and the other method indicated that this could be due to reduced activity of broilers without BFSL at the end of the day. Increased activity could enhance broilers’ quality of life, and future research is necessary to determine how benefits of providing dried larvae can maximized.

**Abstract:**

Welfare issues in broilers include inactivity and poor leg health. Activity can be stimulated by providing whole dried Black Soldier Fly Larvae (BFSL), but it is unknown whether this effect changes over time. Therefore, effects of BSFL provisioning on broiler activity per minute were assessed in detail. Additionally, the use of pixel changes as a proxy for broiler activity was explored. Broilers were housed in groups of 28 (n = 4 pens, <33 kg/m^2^). Dried BSFL were scattered through the pen of treatment groups at 08:00, 11:00, 14:00, and 17:00. Activity was assessed per minute both manually using scan sampling, and automatically as percent pixel changes for 30 min around BSFL provisioning, and at 14, 21 and 28 days of age. Both methods were moderately positively correlated and showed that BSFL provisioning increased activity. Activity as assessed by manual observations decreased at 17:00, at 21, and 28 days in both groups. The pixel changes indicated that this could be due to low activity levels in the control group. Using pixel changes seems to be a promising, timesaving tool to score broiler activity, but future research is necessary to validate this method and determine how high activity can be maintained over time.

## 1. Introduction

Fast-growing broilers in barren housing conditions may experience various welfare problems such as poor leg health [1,2]. The selection-driven rapid growth rate of broilers, one of the risk factors of leg problems, is often accompanied by decreased activity as their high weight makes it difficult to move [2]. The combination of decreased activity and poor litter quality in barren housing systems gives rise to lesions in contact areas such as the breast and legs [1,2]. Vice versa, poor leg health is often painful and hence associated with impaired activity and negative broiler welfare [2]. It is thus not surprising that Western-based European citizens perceive current broiler welfare to be very poor [3,4]. One approach to better broiler welfare is to increase their expression of natural behavior and general activity, for example by providing live larvae, such as Black Soldier Fly Larvae (*Hermetia illucens*, BSFL) [5,6]. Apart from providing welfare benefits, BSFL have a high nutritional value, efficient feed conversion rate, and using BSFL as dietary ingredient for broilers has potential environmental benefits when compared to conventional protein sources used in broiler feed [7,8,9,10]. For example, valorizing waste streams by utilizing them as insect (including BSFL) feed is confirmed to be one of the best strategies for sustainable feed production [7]. Hence, providing BSFL has potential to contribute to a more sustainable broiler meat production, while fulfilling the societal responsibility to improve broiler welfare.

While offering live BSFL has been shown to improve broiler welfare by increasing activity and reducing leg health problems [5,6], the provisioning of live larvae poses a risk of pathogenic contamination, which can endanger feed and food security. For example, residues of *Salmonella* sp., *Bacillus cereus* and *Eimeria tenella* were found in BSFL when these pathogens were present in their feed culture [11,12]. Additionally, BSFL fat can contain the fungi *Aspergillus* spp. and *Cryptococcus neoformans* [13], which may cause respiratory infections [14,15]. Washing the larvae is insufficient for the total removal of pathogens [12], but drying BSFL can significantly reduce the number of pathogens [16,17]. Drying reduces for example the presence of yeasts [16], *Escherichia coli* counts (−4 log, [17]) and makes pathogenic species of *Listeria* non-detectable [16]. Apart from potential feed safety risks, the provisioning of live larvae is challenging as diapause has to be induced by cool temperatures in order to keep the BSFL in their larval stage for periods up to one week. Drying can also prevent spoilage and degradation and extend the expiration date for up to one year [17]. Drying BSFL before utilizing them as broiler feed and enrichment may, therefore, not only reduce health risks but could also have a practical advantage compared to using live larvae.

Several product features of larvae are altered by the drying process [17], which seem to make dried larvae less attractive than live ones [18]. Despite this, a recent study showed that dried BSFL still benefitted broiler welfare, as scattering these in the litter for four times a day increased broiler activity and reduced the occurrence of footpad dermatitis as compared with a control group that did not receive an BSFL feedstuffs [18]. However, this previous study lacks a detailed exploration of activity patterns surrounding the BSFL provisioning and did not provide insights in possible differences in effects throughout the day. The first aim of this study was therefore to assess the effects of dried BSFL provisioning on the broilers’ activity per minute at four different provisioning periods a day and at different ages. Detailed insights in broilers’ activity during BSFL provisioning periods might create a deeper understanding of the effects and could help to maximize the potential of using dried BSFL as environmental enrichment for broilers.

Analyzing behavior on a detailed level can be time-intensive and training is needed in order to obtain proficiency [19,20]. It is therefore of interest to explore novel and time-efficient methods that can measure specific aspects of animal behavior that are often of interest, such as movement. For this reason, a second aim of this study was to compare automatically measured pixel changes and manual observations for mapping broiler activity.

## 2. Materials and Methods

The broilers were part of a larger experiment investigating the effects of different forms of BSFL and different manners of provisioning on broiler welfare published elsewhere [18]. Unless mentioned otherwise, the same conditions apply to this paper. This study took place according to the Dutch law on animal experiments. It was approved by the Animal Care and Use Committee of Wageningen University and Research.

### 2.1. Animals and Housing

A total of 308 male Ross broilers (n = 112) were included in this study. This study started one day post-hatch and continued until broilers were 35 days of age. All broilers received routine vaccinations. For this study, part of the broilers from two treatment groups from the larger experiment were compared: the control (C), which did not receive any BSFL, and the dried scattered (D) group, for which 8% of the expected dry matter (DM) intake consisted of dried BSFL that were scattered around the whole pen by hand in four equal portions a day (at 08:00, 11:00, 14:00, and 17:00 h). The dried BSFL were supplied by Bestico B.V. (Berkel, The Netherlands) and were stored at 10–12 °C near the experimental room.

The broilers were housed at semi-commercial densities (up to 33 kg/m^2^) in a commercial research facility (ForFarmers broiler research farm, Bathmen, the Netherlands). Each pen (n = 4) measured 1.45 × 1.45 m and housed 28 broilers. The pens contained a layer of wood shavings and the animals had ad libitum access to water (six drinking nipples) and feed pellets. Aside from the dried BSFL provisioning in the D group, all broilers received standard starter (day 1–9), grower (day 9–27) and finisher (day 27–35) diets. All diets were balanced for protein and energy intake (See Supplementary Tables S1–S3 in [18]). Pens were separated with panels that had a webbed structure and allowed the broilers to have visual, but not tactile, contact with other pens. The lighting schedule was 23 L:1 D on day 1–2, 20 L:4 D on day 3–7, 18 L:6 D on day 8–33 and 20 L:4 D on day 34–35. The temperature was 34 °C during the first two days, after which it was gradually decreased to 20 °C on day 35.

### 2.2. Experimental Setup, Data Collection and Preparation

**Figure 1:**
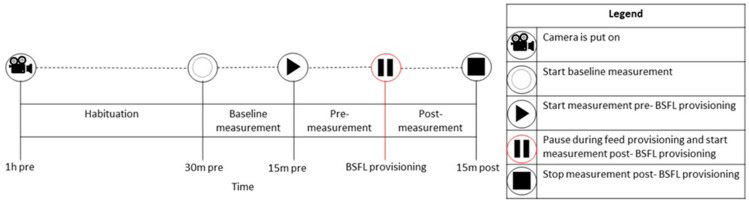
An overview of the data collection with the camera observations related to activity analysis. Video images during BSFL provisioning were discarded (here referred to as Pause, usually a few sec) to avoid noise in the pixel changes as the hand that scattered the larvae was visible.

The behavior of broilers in the two C and in two D pens was collected by placing four cameras (Hikvision DS-2CD2043G2-I 2.8 mm, resolution 2688 × 1520 and frame frequency 25 fps) on statives (Figure S1). Video material of broiler behavior was collected on day 14, 21 and 28. The broilers were left undisturbed (aside from the larvae provisioning itself) from one hour before provisioning onwards and were filmed 30 min pre- and 15 min post-BSFL provisioning (08:00, 11:00, 14:00 and 17:00, Figure 1). The 30 min pre- BSFL provisioning included the baseline measurement (15 min) and the pre-measurement (15 min, Figure 1).

Locomotor activity (movement) of broilers in percent pixel change over total pen area was assessed in EthoVision XT 10 (Noldus Information Technology B.V., Wageningen, The Netherlands) using the grey scaling method and according to Fraess et al. [21]. Hence, each pixel got a value ranging from 0 for black to 255 for white. The pixel value changed when a broiler moved. The more movement there was, the more pixel alterations were detected. The gray scale values of all pixels were compared with the previous frame to determine the percentage of changed pixels between the two. Detection range was automatically set to a maximum of 255 and a compression artefacts filter was applied. Each pen was analyzed individually as a separate arena. Ethovision XT 10 had a sample rate of maximum 25,000 samples per second. Mean number of pixel changes per minute between 30 and 15 min before provisioning within a pen was set as a baseline for this pen reflecting normal activity levels. From this value, the mean percentage change per minute during 15 min pre- and 15 min post the provisioning of dried BSFL was calculated by:

$$\mathrm{Mean}\;\mathrm{percentage}\;\mathrm{pixel}\;\mathrm{change}\;\mathrm{per}\;\mathrm{minute}=\left(\frac{\mathrm{observed}\;\mathrm{number}\;\mathrm{of}\;\mathrm{pixel}\;\mathrm{changes}-\mathrm{baseline}}{\mathrm{baseline}}\right)\times 100$$

Additionally, broiler behavior was scored manually using the ethogram described in Fraess et al. [21]. Behavior was scored for 15 min pre- and post-provisioning BSFL using 1-min instantaneous scan sampling. Per minute, behavior of all 28 broilers in the pen was noted, which took approximately 30 s. The percentage of broilers per pen performing resting behavior (sitting without movement) or active behavior (all other behaviors) was annotated and analyzed per minute. Intra-observer reliability for the manually recorded activity per minute was tested by having the same observer re-score the first 30 observations (minutes) after conducting all observations. Bivariate correlations between investigated variables were analyzed using a two-tailed significance test applying a Pearson correlation coefficient in IBM SPSS Statistics 28.0 (IBM Corp., Armonk, NY, USA). The second time that the first observations were scored was related to the first time the scoring was performed by the observer (r = 0.79, 95%CI [0.60, 0.90], *p* < 0.001), indicating a strong consistency in the manually recorded activity per minute [22].

### 2.3. Statistical Analysis

All data were analyzed using IBM SPSS Statistics 28.0 (IBM Corp., Armonk, NY, USA). An effect was considered significant when *p* ≤ 0.05.

#### 2.3.1. Activity

The percentage of pixel changes per minute and the manually recorded activity per minute before and after providing dried BSFL were analyzed using general linear mixed models separately for each day. The models had a variance components (VC) covariance structure. The model included the fixed effect of treatment (controls, C, vs. dried BFSL, D), the provisioning period (pre- or post-BSFL provisioning) and the provisioning hour (8:00, 11:00, 14:00 and 17:00 h). In addition, the model included all interactions between these parameters as fixed effects. The three-way interaction was never significant. Furthermore, the model included a random effect for pens for the different pre- or post-BSFL provisioning periods. Lastly, the model included a random effect for pens for the different provisioning hours and periods. To achieve normality a ln(y + 100) transformation was applied on the variable pixel changes per minute at 14, 21 and 28 days of age. Assumptions for the linear model were met for the remaining variables. Post hoc comparisons of significant effects were performed using a Bonferroni correction.

#### 2.3.2. Correlation between Variables

Correlations between pixel changes per minute (log transformed prior analysis) and manually recorded activity per minute were analyzed using a two-tailed significance test applying a Pearson correlation coefficient.

## 3. Results

In this section, the differences between D-broilers (which received larvae) and C-broilers (which did not receive larvae) will be presented.

### 3.1. Activity at 14 Days of Age

#### 3.1.1. Manually Recorded Activity per Minute

**Figure 2:**
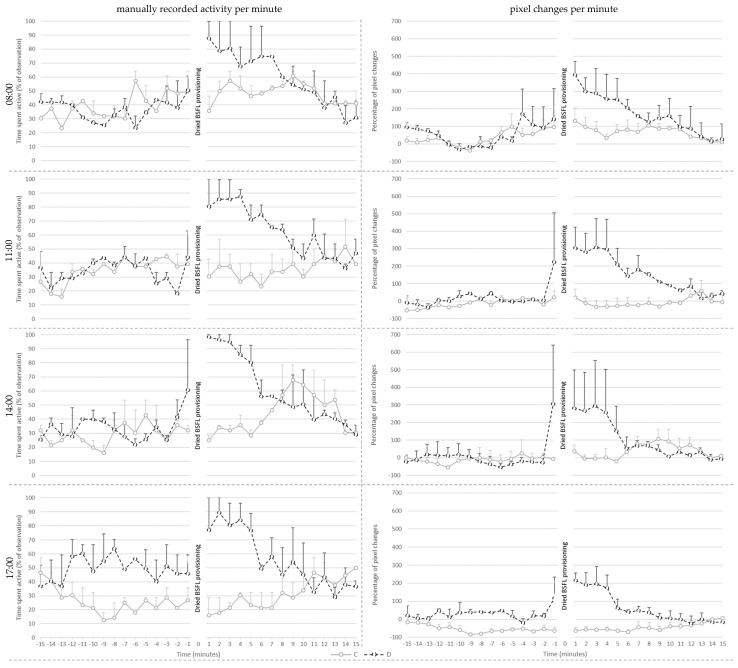
Mean ± SE of the manually recorded activity per minute (**left**) and the mean percentage of pixel changes per minute (**right**) for the control (C) and treatment (D) groups shown per provisioning hour at 14 days of age.

Manually recorded activity per minute on day 14 (Figure 2) was affected by treatment (*p* < 0.001), pre- or post-provisioning period (*p* < 0.001) and their interaction (*p* = 0.021). Post hoc analysis showed that manually recorded activity did not differ between C and D broilers before larvae provisioning. Post-larvae provisioning, however, D broilers showed more active behaviors than before delivery of the larvae (*p* < 0.001), whereas post-larvae provisioning activity of C broilers did not differ from that before the larvae provisioning period. As a consequence, D broilers were more active than the C broilers after larvae provisioning (*p* < 0.001). There were no effects of provisioning hour and its interaction with treatment.

#### 3.1.2. Percentage Pixel Changes per Minute

Percentage pixel changes per minute (Figure 2) was affected by treatment (*p* < 0.001), hour (*p* < 0.001), and their interaction (*p* = 0.049). Post hoc analysis showed that D broilers had a higher percentage of pixel changes per minute compared to C broilers at 11:00 (*p* = 0.02), and at 17:00 (*p* < 0.001). The values of the D broilers did not differ between different provisioning hours. However, C broilers had fewer percent pixel changes at 17:00 compared to at 08:00 (*p* < 0.001) and at 14:00 (*p* = 0.025). Additionally, the percentage of pixel changes was affected by pre- or post-larvae provisioning period (*p* < 0.001), with higher levels post-than pre-larvae provisioning, irrespective of treatment as the interaction between treatment and period was not significant.

### 3.2. Activity at 21 Days of Age

#### 3.2.1. Manually Recorded Activity per Minute

**Figure 3:**
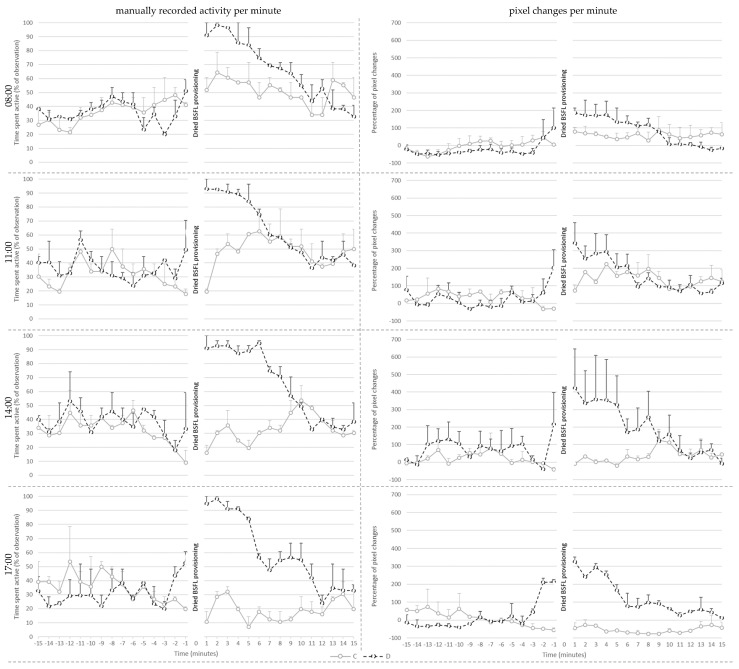
Mean ± SE of the manually recorded activity per minute (**left**) and the mean percentage of pixel changes per minute (**right**) for the control (C) and treatment (D) groups shown per provisioning hour at 21 days of age.

Manually recorded activity per minute on day 21 (Figure 3) was affected by treatment (*p* < 0.001), pre- or post-provisioning period (*p* < 0.001) and their interaction (*p* < 0.001). Post hoc analysis showed that manually recorded activity did not differ between C and D broilers before the larvae provisioning period. Post-larvae provisioning, however, D broilers showed more active behaviors than before delivery of the larvae (*p* < 0.001), whereas post-larvae provisioning activity of C broilers did not differ from that before the larvae provisioning period. As a consequence, D broilers were more active than the C broilers after larvae provisioning (*p* < 0.001).

Additionally, active behavior was affected by the hour in which the dried larvae were provisioned (*p* = 0.016), irrespective of treatment. Post hoc analysis showed that broilers showed more active behavior during the provisioning hour at 8:00 when compared to 17:00 (*p* = 0.016). The values of the broilers did not differ between other provisioning hours.

#### 3.2.2. Percentage Pixel Changes per Minute

Percentage pixel changes per minute on day 21 (Figure 3) was affected by treatment (*p* < 0.024), pre- or post-provisioning period (*p* = 0.002), and their interaction (*p* = 0.014). Post hoc analysis showed that the percentage of pixel changes did not differ between C and D broilers before larvae provisioning. Post-larvae provisioning, however, D broilers showed more pixel changes than before delivery of the larvae (*p* < 0.001), whereas post-larvae provisioning activity of C broilers did not differ from that before larvae. As a consequence, D broilers had higher levels of pixel changes than the C broilers after the larvae provisioning period (*p* = 0.002).

In addition, the percentage of pixel changes was affected by provisioning hour (*p* = 0.006), and the interaction of treatment and the provisioning hour (*p* = 0.031). Post hoc analysis showed that D broilers had a higher percentage of pixel changes per minute compared to C broilers at 17:00 (*p* = 0.002). The values of the D broilers did not differ between different provisioning hours. However, C broilers had fewer percent pixel changes at 17:00 compared to at 11:00 (*p* = 0.002).

### 3.3. Activity at 28 Days of Age

#### 3.3.1. Manually Recorded Activity per Minute

**Figure 4:**
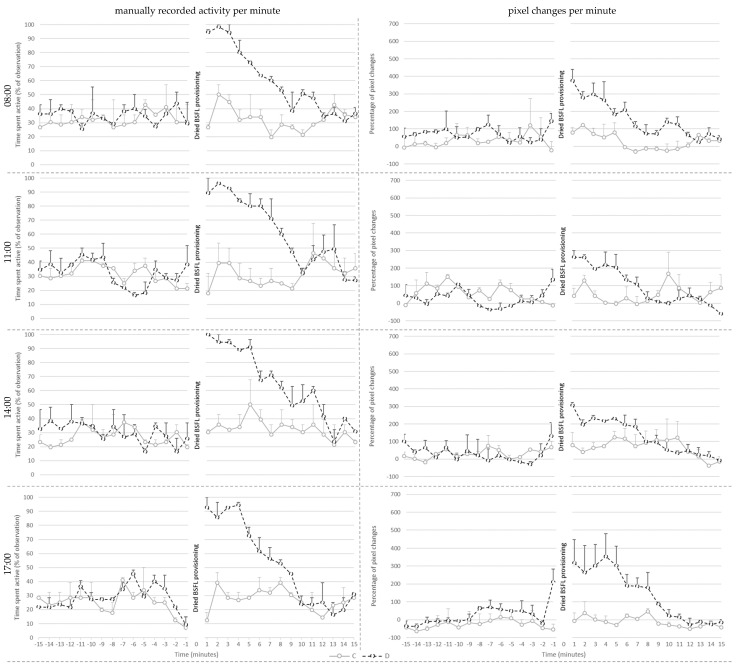
Mean ± SE of the manually recorded activity per minute (**left**) and the mean percentage of pixel changes per minute (**right**) for the control (C) and treatment (D) groups shown per provisioning hour at 28 days of age.

Manually recorded activity per minute on day 28 (Figure 4) was affected by treatment (*p* < 0.001), pre- or post-provisioning period (*p* < 0.001) and their interaction (*p* < 0.001). Post hoc analysis showed that manually recorded activity did not differ between C and D broilers before larvae provisioning. Post-larvae provisioning, however, D broilers showed more active behaviors than before delivery of the larvae (*p* < 0.001), whereas post-larvae provisioning activity of C broilers did not differ from that before larvae. As a consequence, D broilers were more active than the C broilers after the larvae provisioning period (*p* < 0.001).

Additionally, active behavior was affected by the hour in which the dried larvae were provisioned (*p* = 0.006), irrespective of treatment. Post hoc analysis showed that broilers showed more active behavior during the provisioning hour at 8:00 when compared to 17:00 (*p* = 0.01) and at 11:00 (*p* = 0.024). The values of the broilers did not differ between other provisioning hours.

#### 3.3.2. Percentage Pixel Changes per Minute

Percentage pixel changes per minute (Figure 4) was affected by treatment (*p* = 0.002), hour (*p* = 0.024), and their interaction (*p* = 0.027). Post hoc analysis showed that D broilers had a higher percentage of pixel changes per minute compared to C broilers at 08:00 (*p* = 0.013), and at 17:00 (*p* < 0.001). The values of the D broilers did not differ between different provisioning hours. However, C broilers had fewer percent pixel changes at 17:00 compared to at 11:00 (*p* = 0.007) and at 14:00 (*p* = 0.01). Additionally, the percentage of pixel changes was affected by pre- or post-larvae provisioning period (*p* = 0.006), with higher levels post-than pre-larvae provisioning.

### 3.4. Relation Pixel Changes per Minute and Manually Recorded Activity per Minute

**Figure 5:**
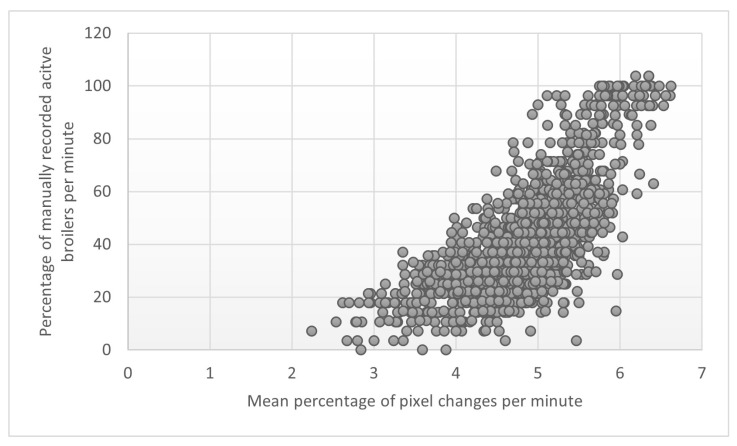
Scatterplot of the relation between mean percentage pixel changes per minute (log transformed, *x*-axis) and manually recorded activity per minute (as a % of the observation, *y*-axis) around larvae provisioning and of all observation days combined.

There was a moderate positive correlation between the manually recorded activity per minute and the per cent pixel changes per minute in the 15 min before and after the dried BSFL provisioning (r = 0.72, 95%CI [0.69, 0.74], *p* < 0.001), indicating that the percentage of manually recorded activity per minute increases when the percentage of pixel changes per minute does so too (Figure 5).

## 4. Discussion

This study aimed to map out the dynamics of broiler activity around dried Black Soldier Fly Larvae provisioning at four times a day, as improved activity may contribute to enhanced broiler welfare [5,6,18]. To that aim, activity of broilers provided with dried larvae (D) was compared to that of a control group (C). In addition, the use of manual observations with 1-min scan sampling and automatically detected pixel changes as an indicator for broiler activity was compared.

The percentage of manually recorded activity per minute was moderately correlated with the percentage of pixel changes per minute. Both measurements revealed differences in activity between C and D broilers, but the exact effects found varied somewhat between the two. When using manual observations, an increase in activity of the D broilers was observed after larvae provisioning on all observation days (day 14, 21 and 28). A similar effect was found for pixel changes on day 21, although on day 14 and 28 only a main effect of period (before vs. after provisioning of larvae) was found, irrespective of treatment, possibly reflecting that also C broilers slightly increased their activity after the provisioning of BFSL to the D broilers. Activity patterns around feeding BFSL varied between hours of the day, with lowest activity at the last provisioning moment. Manually recorded activity and percent pixel changes per minute slightly differed in revealing effects of dried larvae provisioning on activity of broilers at different hours. Assessing pixel changes should, therefore, be considered complementary to manual behavioral assessments as it provides additional information on a more detailed level.

### 4.1. Treatment and Time Effect on Broiler Activity

The effect of BSFL provisioning on activity appeared to be influenced by the provisioning hour as activity shown in the manual scans was lowest at 17:00 at 21 and 28 days of age, irrespective of treatment. Near the onset of the dark period the feeding behavior of broilers may decline [23,24]. It is possible that broilers decreased feed driven activity during the BSFL provisioning period at 17:00, as this period is closest to the onset of the dark period when compared to the other, earlier, periods. Another explanation for the low activity at 17:00 might be that the D broilers were already satiated after receiving BSFL in the first three provisioning periods. However, this is not completely in line with research investigating the use of live BSFL as environmental enrichment for broilers that suggested that feeding BSFL for four times a day is more effective in bettering broiler than lower frequencies (i.e., two times a day, [5]).

However, unlike activity measurements based on manual observations, the effects of provisioning hour on the pixel changes were dependent on treatment, indicating that particularly activity levels of the C broilers were low at the last provisioning hour of the day. In contrast, pixel changes in D pens were not different between the four BSFL provisioning hours. Future research could investigate if variating dried BSFL provisioning periods could be used to stimulate broiler activity in the afternoon. Activity is beneficial for welfare as it can, for example, benefit leg health [5,6,18]. Welfare could, however, be influenced by other factors such as management, future research should take this into account.

When visually observing the data, D-broilers seemed to show anticipatory behavior prior to BSFL provisioning (Figure 2, Figure 3 and Figure 4). This anticipatory behavior, likely unintentionally induced by caretakers entering the room, could further stimulate activity in broilers provided with dried larvae, on top of the foraging for the larvae after dried BSFL provisioning. Possibly, anticipation could be extended and used to prolong the active period around larvae provisioning, providing that frustration can be avoided. The anticipatory increase in activity is particularly reflected in the visual representation of the percent pixel changes. It is unknown to what extent anticipatory behavior contributed the absence of the interaction between treatment and provisioning period (pre- vs. post-larvae provisioning).

Visually, C-broilers seemed to have a small increase in activity post-the BSFL provisioning period. This was not statistically supported by the manually recorded activity as solely the D broilers showed a significant increase. It could be speculated that this slight increase in activity in C broilers contributed to the absence of an interaction between treatment and provisioning method for the percent pixel changes on day 14 and 28. Possibly, the C broilers were aroused by the caretakers walking around to provide larvae to the other groups, and/or by the resulting changes in behavior of these groups, particularly the ones in neighboring pens. Chickens are highly social animals, using referential communication [25] and it has been found that feeding behavior of a single broiler promotes group feeding behavior, which indicates that feeding behavior is socially facilitated [26]. The stimulation of group feeding behavior in the absence of the preferred larvae might, however, also induce frustration. More research is necessary to be more conclusive about the presence, role and degree of anticipatory behavior and social facilitation around provisioning of the dried BSFL. Future studies may investigate the impact of variation in the opportunity of chickens to see, hear and smell each other during the feed provisioning. If behavioral and/or emotional transfer is present to a high degree and frustration is not induced, one may argue that not every broiler needs to receive dried BSFL in order to increase broiler activity and thereby welfare. If this is the case, farmers could better this aspect of broiler welfare by feeding relatively low quantities which makes this form of enrichment financially more advantageous.

### 4.2. Comparing Pixel Changes and Manual Observations as Measurements of Activity

There was a moderately positive relationship between pixel changes and manual observations of activity [27]. This is in line with the study of Fraess et al. [21], in which it was found that pixel changes could be used to detect a feeding moment and differentiate between different layer breeds. The exact effects of treatment on activity around larvae provisioning at different hours varied somewhat between both methods. When assessing pixel changes, more subtle differences between treatment groups at different hours were detected as the interaction between treatment and hour was always significant. On the other hand, treatment differences in response to larvae provisioning seemed to be revealed to a higher degree when analyzing the manual recordings, as the interaction between treatment and pre- and post-provisioning period was always significant for this variable.

It seems that the pixel changes assessment might have missed some activity if manual scoring is regarded the gold standard. There are, however, several other possible explanations for the different effects found by both methods. Firstly, a baseline was used to calculate the percentage of pixel changes. Variations in the values that are set as the baseline could influence the results shown in pixel alterations while the results of the manual scoring were not influenced by a baseline. Second, manual scoring might be more accurate for detecting small movements that may not be reflected in the change in many pixels [21], such as ground pecking. Pixel changes reflect movement. However, the manual scoring method solely assessed whether broilers were active or not, and no distinction was made in the degree of activity. Third, pixel changes were averaged over time, whereas manual observations were based on scans. The continuously assessing nature of the software analysis is able to assess frequency and magnitude differences in a way that 30 s scan sampling could not. Further validation of the found results with a bigger sample size and in variating settings (e.g., intensive and extensive poultry farming systems) can contribute to a simple and time efficient method of scoring broiler activity.

## 5. Conclusions

The results suggest that providing whole dried BSFL could be a beneficial addition to the environment of broilers as it stimulates their activity levels throughout the day and across weeks. This effect could be influenced by the provisioning hour. In addition, assessing the percentage of pixel changes seems promising and can be a time-saving tool to score active behavior, and it could be considered complementary to manual behavioral assessments as it provides additional information on a more detailed level.

## Data Availability

Data will be made available on request.

## Supplementary Materials

The following supporting information can be downloaded at: https://www.mdpi.com/article/10.3390/ani13132200/s1, Figure S1: video camera statives.

## References

[1] De Jong I.C., van Harn J., Gunnink H., Hindle V.A., Lourens A. (2012). Footpad Dermatitis in Dutch Broiler Flocks: Prevalence and Factors of Influence. Poult. Sci..

[2] Bessei W. (2006). Welfare of Broilers: A Review. Worlds. Poult. Sci. J..

[3] Vanhonacker F., Verbeke W., Van Poucke E., Tuyttens F.A.M. (2008). Do Citizens and Farmers Interpret the Concept of Farm Animal Welfare Differently?. Livest. Sci..

[4] Verbeke W. (2009). Stakeholder, Citizen and Consumer Interests in Farm Animal Welfare. Anim. Welf..

[5] Ipema A.F., Gerrits W.J.J., Bokkers E.A.M., Kemp B., Bolhuis J.E. (2020). Provisioning of Live Black Soldier Fly Larvae (*Hermetia illucens*) Benefits Broiler Activity and Leg Health in a Frequency- and Dose-Dependent Manner. Appl. Anim. Behav. Sci..

[6] Ipema A.F., Bokkers E.A.M., Gerrits W.J.J., Kemp B., Bolhuis J.E. (2020). Long-Term Access to Live Black Soldier Fly Larvae (*Hermetia illucens*) Stimulates Activity and Reduces Fearfulness of Broilers, without Affecting Health. Sci. Rep..

[7] Smetana S., Palanisamy M., Mathys A., Heinz V. (2016). Sustainability of Insect Use for Feed and Food: Life Cycle Assessment Perspective. J. Clean. Prod..

[8] Onsongo V.O., Osuga I.M., Gachuiri C.K., Wachira A.M., Miano D.M., Tanga C.M., Ekesi S., Nakimbugwe D., Fiaboe K.K.M. (2018). Insects for Income Generation Through Animal Feed: Effect of Dietary Replacement of Soybean and Fish Meal with Black Soldier Fly Meal on Broiler Growth and Economic Performance. J. Econ. Entomol..

[9] Akhtar Y., Isman M.B. (2018). Insects as an Alternative Protein Source. Proteins in Food Processing.

[10] Parodi A., Ipema A.F., Van Zanten H.H.E., Bolhuis J.E., Van Loon J., De Boer I.J.M. (2022). Principles for the responsible use of farmed insects as livestock feed. Nat. Food..

[11] Wynants E., Frooninckx L., Crauwels S., Verreth C., De Smet J., Sandrock C., Wohlfahrt J., Van Schelt J., Depraetere S., Lievens B. (2019). Assessing the Microbiota of Black Soldier Fly Larvae (*Hermetia illucens*) Reared on Organic Waste Streams on Four Different Locations at Laboratory and Large Scale. Microb. Ecol..

[12] Müller A., Wiedmer S., Kurth M. (2019). Risk Evaluation of Passive Transmission of Animal Parasites by Feeding of Black Soldier Fly (*Hermetia illucens*) Larvae and Prepupae. J. Food Prot..

[13] Thrastardottir R., Olafsdottir H.T., Thorarinsdottir R.I. (2021). Yellow Mealworm and Black Soldier Fly Larvae for Feed and Food Production in Europe, with Emphasis on Iceland. Foods.

[14] Akan M., Hazroǧlu R., Ilhan Z., Sareyyüpoǧlu B., Tunca R. (2002). A Case of Aspergillosis in a Broiler Breeder Flock. Avian Dis..

[15] Radwan I.A., Abed A.H., Abd El-Aziz M.M. (2016). Fungal Pathogens Associated with Respiratory Problems in Broiler Chickens. J. Vet. Med. Res..

[16] Larouche J. (2019). Processing Methods for the Black Soldier Fly (*Hermetia illucens*) Larvae: From Feed Withdrawal Periods to Killing Methods. Master’s Thesis.

[17] Saucier L., M’ballou C., Ratti C., Deschamps M.H., Lebeuf Y., Vandenberg G.W. (2020). Comparison of Black Soldier Fly Larvae Pre-Treatments and Drying Techniques on the Microbial Load and Physico-Chemical Characteristics. J. Insects Food Feed.

[18] Ipema A.F., Bokkers E.A.M., Gerrits W.J.J., Kemp B., Bolhuis J.E. (2022). Provision of Black Soldier Fly Larvae (*Hermetia illucens*) in Different Ways Benefits Broiler Welfare and Performance, with Largest Effects of Scattering Live Larvae. Physiol. Behav..

[19] Viscardi A.V., Hunniford M., Lawlis P., Leach M., Turner P.V. (2017). Development of a Piglet Grimace Scale to Evaluate Piglet Pain Using Facial Expressions Following Castration and Tail Docking: A Pilot Study. Front. Vet. Sci..

[20] Stamp Dawkins M. (2008). Observing Animal Behaviour: Design and Analysis of Quantitative Data.

[21] Fraess G.A., Bench C.J., Tierney K.B. (2016). Automated Behavioural Response Assessment to a Feeding Event in Two Heritage Chicken Breeds. Appl. Anim. Behav. Sci..

[22] Hazra A., Gogtay N. (2016). Biostatistics Series Module 6: Correlation and Linear Regression. Indian J. Dermatol..

[23] Shynkaruk T., Classen H.L., Crowe T.G., Schwean-Lardner K. (2019). The impact of dark exposure on broiler feeding behavior and weight of gastrointestinal tract segments and contents. Poult. Sci..

[24] Rodrigues I., Choct M. (2019). Feed intake pattern of broiler chickens under intermittent lighting: Do birds eat in the dark?. Anim. Nutr..

[25] Marino L. (2017). Thinking Chickens: A Review of Cognition, Emotion, and Behavior in the Domestic Chicken. Anim. Cogn..

[26] Collins L.M., Sumpter D.J.T. (2007). The Feeding Dynamics of Broiler Chickens. J. R. Soc. Interface.

[27] Schober P., Schwarte L.A. (2018). Correlation Coefficients: Appropriate Use and Interpretation. Anesth. Analg..

