# Supplementary material for: Comparing Pixel Changes and Manual Observations for Mapping Broiler Activity during Dried Black Soldier Fly Larvae (Hermetia illucens) Provisioning
Source: Animals (Basel). 2023 Jul 5;13(13):2200. doi: 10.3390/ani13132200 (PMC10340013; doi:10.3390/ani13132200)
Supplement: Supplementary file 1 [file animals-13-02200-s001.zip › animals-2461147-supplementary.pdf]

Supplementary Materials

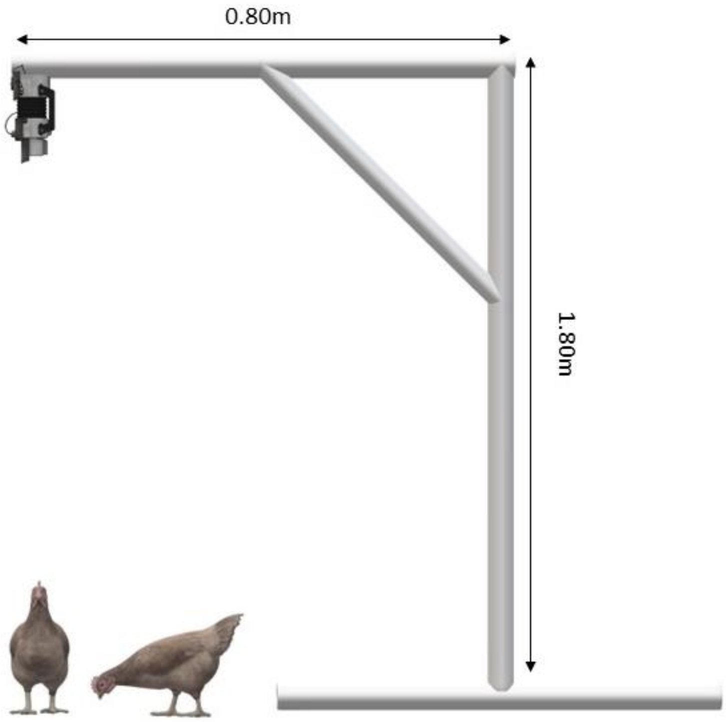

Figure S1. An illustration of the statives on which the camera was placed. The height of the statives was 1.80 m. the horizontal part to which the camera was attached was 0.80 m.
